# The influence of preoperative anxiety on postoperative pain in patients undergoing cardiac surgery

**DOI:** 10.1038/s41598-022-20870-9

**Published:** 2022-10-01

**Authors:** Mercedes Fernández-Castro, José-María Jiménez, Belén Martín-Gil, María-Fe Muñoz-Moreno, Ana-Belén Martín-Santos, Isaías del Río-García, Natán Redondo-Pérez, María López

**Affiliations:** 1 Hospital Clínico Universitario de Valladolid, Valladolid, Spain; 2 University of Valladolid, Valladolid, Spain

**Keywords:** Cardiology, Health care, Medical research

## Abstract

Patients undergoing cardiac surgery represent a challenge in terms of pain management due to multiple factors relating to the patients and to the procedure itself. Our aim was to identify the influence of levels of preoperative anxiety on postoperative pain in patients undergoing cardiac surgery and explore associations between preoperative anxiety, postoperative pain, analgesic requirements, and sex. We present a prospective cohort study of 116 patients undergoing cardiac surgery between January and April 2020. Preoperative anxiety was evaluated using the State-Trait Anxiety Inventory and the amount of morphine needed to keep pain intensity below 4 on the verbal numerical rating scale was recorded for 48 h post-surgery. Given the extracorporeal circulation time, type of surgery and body surface, it was observed that every percentile increase in preoperative state anxiety led to an extra 0.068 mg of morphine being administered. For each extra year of age, the amount of morphine needed decreased by 0.26 mg, no difference was observed between men and women in terms of preoperative anxiety or postoperative analgesics requirements. It may be concluded that in cardiac surgery, postoperative analgesic requirements increased with higher levels of preoperative state anxiety and decreased for every extra year of age.

## Introduction

In patients undergoing cardiac surgery, postoperative pain is one of the main causes for concern. Recent studies show that, during the postoperative period, 47% to 75% of patients experienced pain. This pain is often severe and insufficiently treated^1,2^. Inadequate analgesia during the postsurgical period can lead to adverse cardiovascular responses, such as hypertension, tachycardia, vasoconstriction, haemodynamic instability, and high incidence of postoperative myocardial infarction. This increase in morbidity may interfere with extubation time, resulting in longer hospital stays and higher overall costs^3^. Patients undergoing cardiac surgery pose a challenge when it comes to pain management because of the multiple, diverse characteristics of each patient and procedure, so many tools for evaluating postoperative pain have been validated among cardiac surgery populations^1^.

Significant factors predicting greater sensitivity to postoperative pain from cardiac surgery include: experiencing preoperative pain, feeling anxiety, being younger, and being female^4^. Factors such as optimism, depression, chronic pain (persistent pain for more than three months as a criterion of temporality) and catastrophic pain have also been linked to acute postoperative pain from cardiac surgery^5^. Within strictly surgical factors, time of extracorporeal circulation and type of cardiac surgery affects the overall recovery including postoperative pain^6^.

Research has linked higher levels of preoperative anxiety to greater postoperative pain^5,7,8^. Preoperative anxiety can cause haemodynamic issues during the intraoperative period, increased need for analgesics, and poorer patient satisfaction in the postoperative period^8^. Given this, the recently updated guidelines from the European Society of Anaesthesiology recommend incorporating anxiety assessment into preoperative patient assessments^9^.

One of the most reliable and most widely used tools to measure preoperative anxiety in cardiac surgery is the State-Trait Anxiety Inventory (STAI)^10^. The inventory’s authors identify two types of anxiety: a temporary state or State Anxiety (SA), and a stable latent trait or Trait Anxiety (TA). SA is conceptualised as a transitory emotional state or condition of the human body that is characterised by subjective feelings of tension and apprehension, which are consciously perceived, as well as a hyperactive autonomic nervous system. It can vary over time and fluctuate in intensity. TA is a relatively stable propensity for anxiety, making individuals more likely to perceive situations as threatening and exacerbating their SA^11^.

Studies have shown that women experience higher levels of persistent pain up to a year after cardiac surgery, although they note that this may be linked to a lower intake of analgesics^12^. It has also been suggested that women are more sensitive to pain. Although the reasons for this remain unclear^13^. The considerable differences in men and women’s molecular and cellular genetic mechanisms and systems for processing acute and chronic pain are undoubtedly affected by the interaction between sex and other sociocultural factors, and this is likely to play an extremely important role in experiences of pain^14^.

Postoperative pain is a complex, multifactorial phenomenon and an important prognostic predictor among cardiac surgery patients. The notion of individualised pain management based on associated factors needs further research in order to be applied to the development of tools for predicting who will experience increased pain and need more analgesia, or for the early implementation of interventions to reduce modifiable factors, such as preoperative anxiety^15^.

The relationship between preoperative anxiety and postoperative pain becomes apparent. This study differs from previous publications in that it links preoperative anxiety not only to the degree of pain reported by patients in the immediate postoperative period, but also to the degree of analgesia necessary to keep pain at a mild level.

## Methods

This study aims to identify the influence of levels of preoperative anxiety on predisposition to postoperative pain in patients undergoing cardiac surgery based on analgesic requirements, as well as to explore the relationship between preoperative anxiety, postoperative pain, and sex.

### Study design

This prospective cohort study of patients undergoing cardiac surgery evaluated preoperative anxiety levels as a factor influencing immediate postoperative pain.

### Participants

Patients aged over 18 undergoing elective cardiac surgery at a tertiary hospital run by the Castile and León Regional Health Management Board, Spain, between January and April 2020. The following types of cardiac surgery were covered: coronary revascularisation (a graft using the mammary artery or the saphenous vein) and/or valvular (repair or replacement) surgery. All of them with sternotomy and extracorporeal circulation. Patients who did not consent to participate in the study after receiving information about it were excluded. A total of 13 patients were excluded: 1 exitus occurred in the first 24 h after surgery, 8 patients were unable to perform the preoperative anxiety test due to last minute changes to the surgery schedule, and 4 patients provided incomplete data on postoperative pain. A total of 116 patients who met the inclusion criteria were finally included. The optimal sample size was set at 113 participants with a 95% confidence interval, an accuracy of 0.5 units, and a standard deviation of 2.5 units for mean postoperative pain. The replacement rate was set at 15%.

### Data collection

Data were collected on: (a) sociodemographic variables: age and sex; (b) most common comorbidities and toxic habits in the last month (smokers: more than 5 cigarettes a day and alcoholics: daily consumption of more than 60 g of alcohol in men and more than 40 g for women or the occasional consumption of more than 60 g of alcohol in a single drink.); (c) type of cardiac surgery (coronary, valvular, or mixed), and extracorporeal circulation time (minutes); (d) psychological variables: SA/TA level (score from 0 to 60 for each subscale); (e) analgesic requirements in the postoperative period: milligrams of morphine hydrochloride required to maintain pain below 4 on the verbal numerical rating scale (vNRS), which ranges from 0 (no pain) to 10 (maximum pain) and, (f) body surface calculated with height and weight. About analgesic requirements, it is known that weight or body surface area makes a difference in the morphine dosage recommendations. The drugs administered during surgery (fentanyl and etomidate or propofol and rocuronium bromide) were not taken into consideration, as they were used according to protocol.

### Measures

In the 24 h prior to surgery, patients in the cardiac surgery inpatient unit who met the inclusion criteria were informed of the study objectives and asked for their informed consent. After they had signed the informed consent form, the patients completed the Spanish version of the State-Trait Anxiety Inventory (STAI). This psychometric questionnaire comprises two scales measuring different facets of anxiety: state and trait anxiety. The STAI contains 40 items. Half of the items belong to the State subscale, which is made up of phrases describing how the respondent feels at that specific moment. The other half belong to the Trait subscale, which encompasses phrases describing how the respondent usually feels. The Spanish adaptation of the questionnaire has good internal consistency at 0.90–0.93 (Cronbach alpha) for the State subscale and 0.84–0.87 (Cronbach alpha) for the Trait subscale. In the Spanish version, the direct scores corresponding to the sum of the items have been converted into centile and sten scores, which are obtained according to the sex and age indicated by the authors of the Spanish adaptation^16^. Centile scores were used in this study. Patients were asked to respond to the items on the SA scale, which ranges from 0 (not at all) to 3 (a lot), and the TA scale, which runs from 0 (almost never) to 3 (almost always). The minimum direct score was 0 and the maximum was 60 for both subscales, and this score was then converted into the corresponding centile. Two members of the research team administered the questionnaires in all cases, giving patients the same instructions for completing the questionnaire to reduce potential bias^17^.

After their surgery, patients’ pain was evaluated and monitored in the Cardiac Recovery Unit throughout the first 48 h post-surgery. The first 48 h after surgery were selected as the follow-up period for measuring patients’ pain, as several studies have identified this as the most painful period following cardiothoracic surgery^2,18^.

Evaluation of postoperative pain was carried out once patients had been extubated and were able to verbalise their pain. The vNRS scale was used to measure their pain (0: no pain and 10: maximum pain). Pain intensity was recorded every two hours in two daily shifts: morning (08:00 to 15:00), evening (15:00 to 22:00) and night (22:00 to 08:00). Patients’ sleep was respected during the night shift, with at least two measurements taken at 00:00 and 06:00. The maximum pain values recorded in each shift throughout the course of the 48-h monitoring period were used for the study.

The protocol for managing cardiac surgery patients’ postoperative pain at the Cardiac Recovery Unit offered two options based on medical criteria: (A) Analgesic combination of 1 g of paracetamol every 8 h and 50 mg of dexketoprofen every 8 h administered alternately and (B) 1 g of paracetamol every 8 h and 2 g of metamizole magnesium every 8 h administered alternately. In addition, whenever patients expressed pain exceeding 4 on the vNRS scale, IV boluses containing 3 mg of morphine hydrochloride were administered as ‘rescue doses’. The ‘analgesic requirements’ variable was measured by calculating the total milligrams of morphine hydrochloride required by patients over the 48-h period.

### Ethical approval

The study was conducted in line with the principles stated in the Declaration of Helsinki. The permission to conduct the study was approved by the Clinical Research Ethics Committee of the East Valladolid Health Area (Reference Number: GR-19-1253).

### Statistical analysis

Data were analysed using the IBM SPSS Statistics for Windows, Version 24.0. Armonk, NY: IBM Corp. Quantitative variables were expressed as means and standard deviations. Qualitative variables, in terms of their frequency distribution. The assumption of normality was assessed using the Kolmogorov–Smirnov test. Associations between qualitative variables were explored using Pearson’s chi-squared test. When more than 20% of the cells had expected values under 5, Fisher’s exact test or the likelihood-ratio test for variables with more than two categories was used. Student’s *t*-test was used to compare two groups of quantitative values and ANOVA was used to compare more than two groups of quantitative values. To analyse the degree of association between quantitative variables, Pearson’s correlation coefficient was used. A univariate linear regression model was performed to identify the factors linked to the dose of morphine received by each patient. Variables found to be statistically significant at the 0.1 level in the previous analyses were entered into a multivariate linear regression model. The performance of association of the model was calculated with the coefficient of determination R-squared (R^2^). The statistical significance threshold for all tests was set at p < 0.05.

## Results

A sample of 116 patients comprising 68% men and 32% women was analysed. The mean age was 70 years old (standard deviation (SD) = 8.08). 23.3% (n = 27) of the patients underwent coronary cardiac surgery, 55.2% (n = 64) underwent valvular coronary surgery, and 21.6% (n = 25) underwent mixed surgery. The mean extracorporeal circulation time was 107.73 min (SD = 42.42).

The most common comorbidities were, 49.1% (n = 57) experienced high blood pressure and 19.8% (n = 23) diabetes. Only one patient (0.9%) reported suffering from chronic pain (see Table 1).

**Table 1:** Frequencies of comorbidities and toxic habits in the study sample.

	n	%
**Comorbidities**		
High blood pressure	57	49.1
Diabetes	23	19.8
Allergy	10	8.6
Prior cardiac surgery	8	6.9
Chronic obstructive pulmonary disease	8	6.9
Prior cardiac surgery	8	6.9
Obesity	7	6.0
Pulmonary hypertension	5	4.3
Chronic renal failure	5	4.3
Hypothyroidism	4	3.4
Neurological disease	2	1.7
Brain strokes	2	1.7
Liver disease	2	1.7
Asthma	2	1.7
Cancer (last 3 months)	1	0.9
Autoimmune disease	1	0.9
Chronic pain	1	0.9
**Toxic habits**		
Smokers	29	25
Alcoholics	7	6

With regard to preoperative anxiety, the total sample obtained a median of 60 (25th percentile = 40; 75th percentile 75 = 80) for SA, and a median of 30 for TA (25th percentile 25 = 13; 75th percentile 75 = 55).

**Figure 1:**
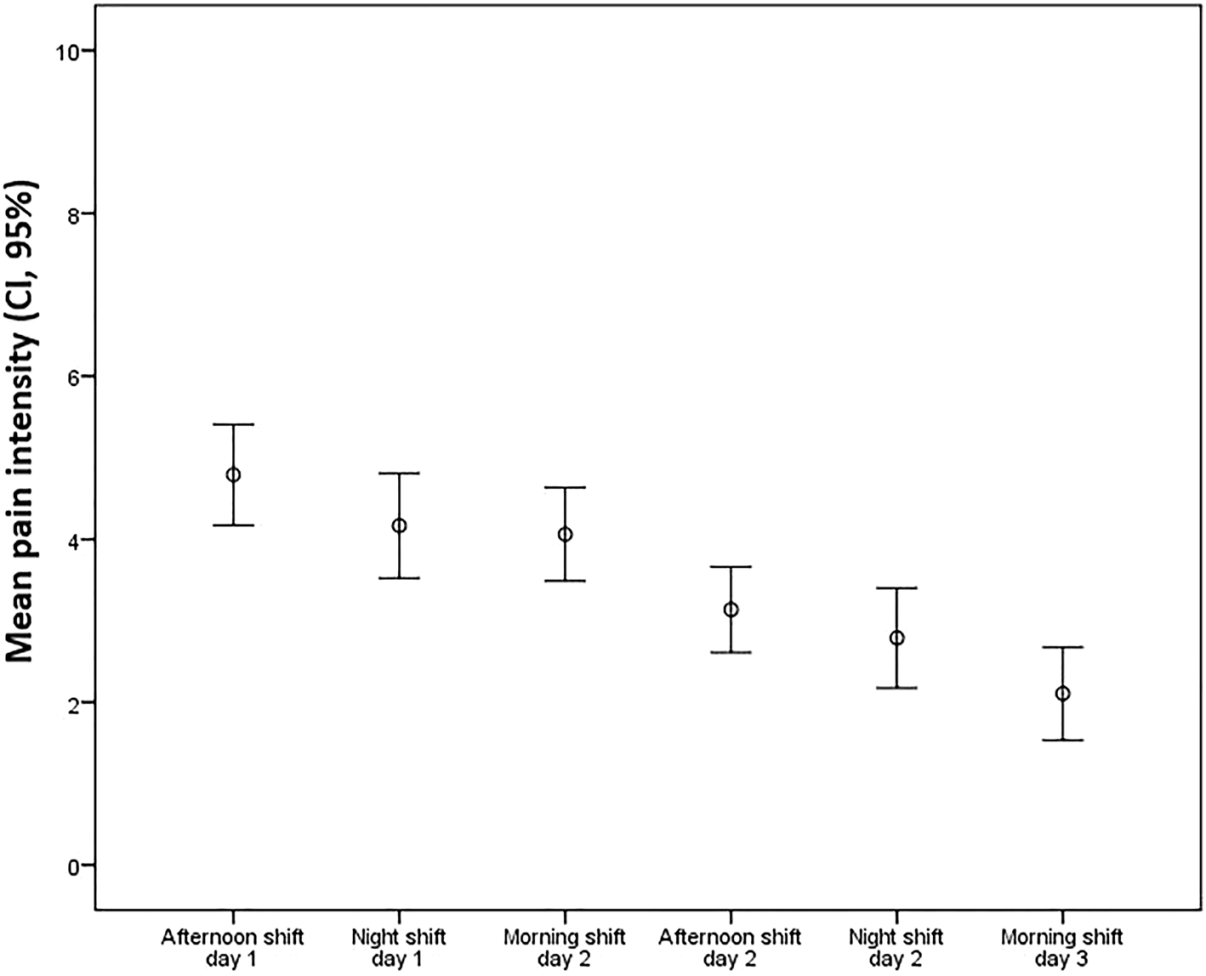
Decrease in mean pain intensity (CI, 95%) over the course of successive shifts in the first 48 h of the postoperative period, according to the verbal numerical rating scale (p < 0.001).

The mean value for postoperative pain was 3.57 (SD = 1.81) on the vNRS scale. The mean dose of morphine needed as a rescue dose was 13.21 mg (SD = 7.59). The pain intensity verbalised by patients decreased significantly throughout the evening, night, and morning shifts in the 48-h follow-up period (p < 0.001) (see Fig. 1).

A comparison of the two different regimes of postoperative pain, either dexketoprofen and metamizole was made. There were no statistical differences with respect to pain intensity and morphine consumption among dexketoprofen and metamizol groups (p > 0.05).

Based on univariate analyses the following variables that were significantly related to postoperative morphine use at a 0.1 level, were entered in the multivariate model: rescue medication corrected for body surface area, extracorporeal circulation time and type of surgery,

Patients with higher levels of preoperative SA were found to need more morphine hydrochloride to keep their pain level below 4 on the vNRS (p = 0.029). On average, every percentile increases in SA required an extra 0.068 mg of morphine hydrochloride in the rescue doses administered. Considering age, every extra year of age reduced morphine hydrochloride requirements by 0.25 mg (p = 0.017). The coefficient of determination (R^2^) obtained in the multivariate linear regression model was 0.77. This result indicates that the percentage of variability explained for pain taking into account milligrams of morphine and state anxiety is good. The results of the multivariate linear regression model can be seen in Table 2.

**Table 2:** Variables linked to milligrams of morphine hydrochloride administered as rescue doses in the postoperative period. Multivariate linear regression model.

	Unstandardised coefficients	Standardised coefficients	p-value	95% confidence interval for B	
	*B*	Beta		Lower limit	Upper limit
(Constant)	23.223		0.057	− 0.664	47.109
State anxiety	0.068	0.252	0.029	0.007	0.129
Age	− 0.257	− 0.270	0.017	− 0.468	− 0.047
Sex (Male as reference)	− 2.636	− 0.160	0.222	− 6.905	1.632
Body surface	3.914	0.111	0.368	− 4.700	12.528
Extracorporeal circulation time (min)	− 0.021	− 0.113	0.306	− 0.062	0.020
Coronary surgery (Mixed surgery as reference)	− 1.474	− 0.084	0.577	− 6.723	3.774
Valvular surgery (Mixed surgery as reference)	− 0.583	− 0.038	0.809	− 5.379	4.213

**Figure 2:**
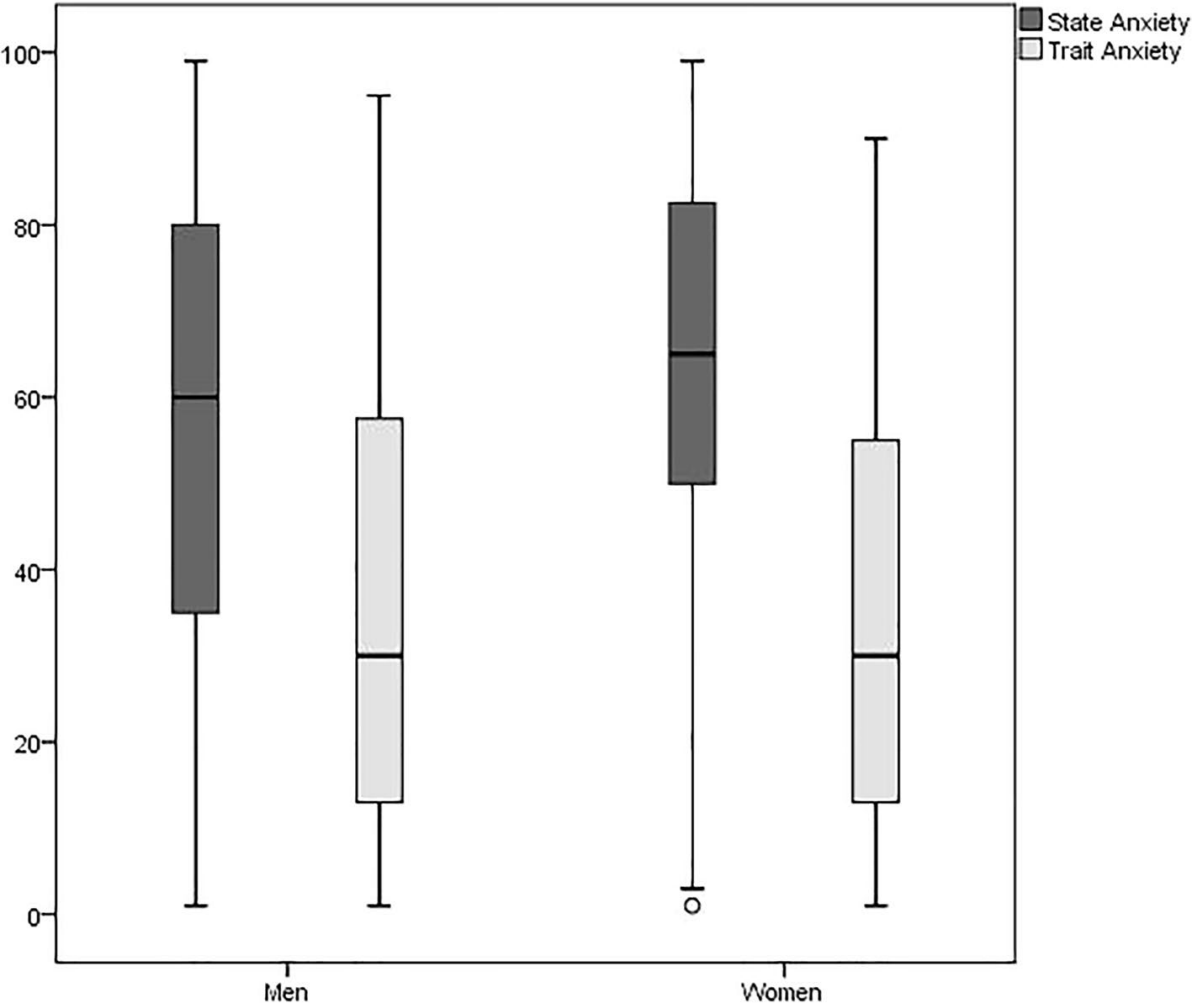
Comparison of median preoperative SA and TA between men and women.

Regarding the analysis of preoperative anxiety by sex, no statistically significant differences were observed between men and women’s levels of preoperative anxiety on the SA or TA subscales p > 0.05) (see Fig. 2).

**Figure 3:**
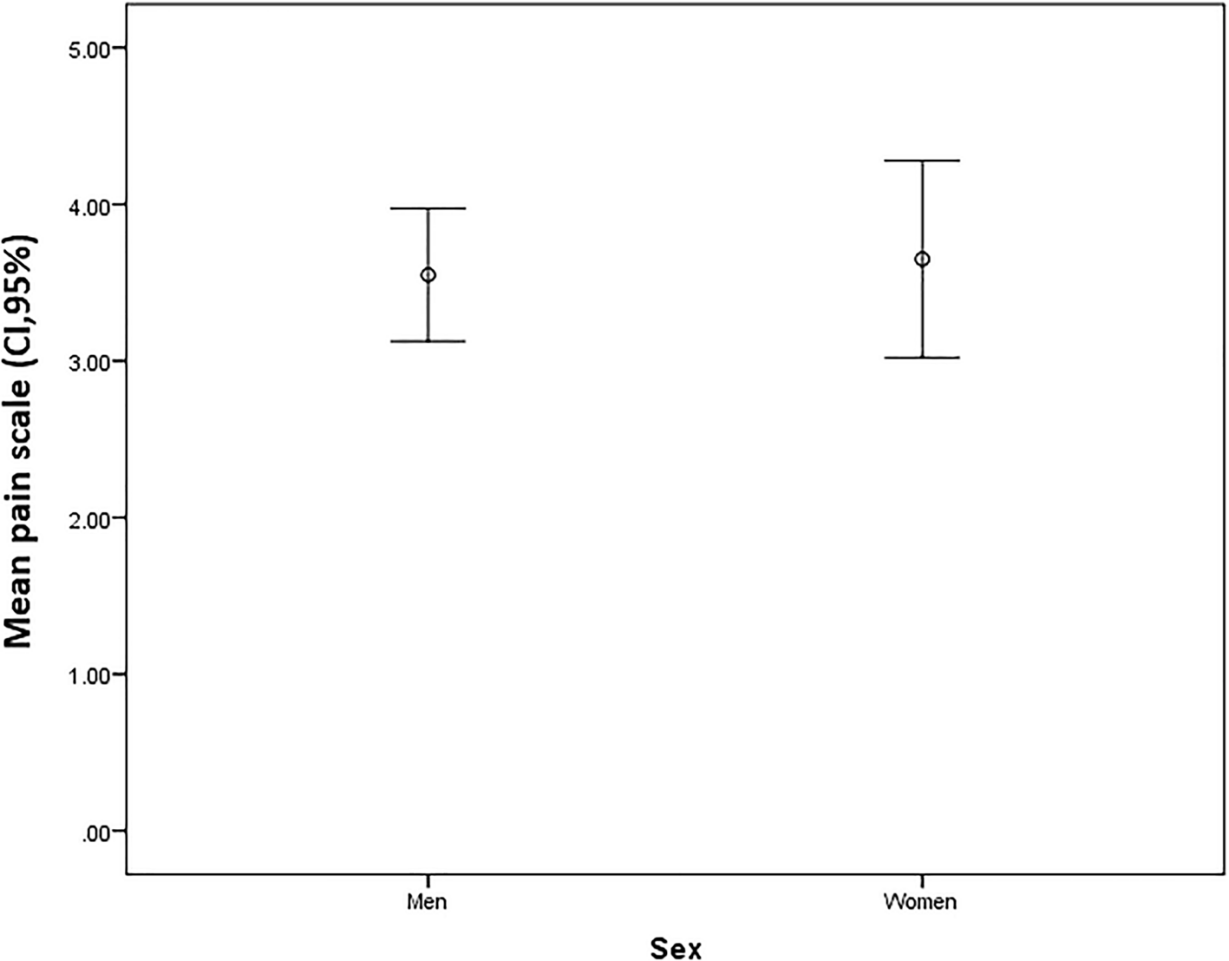
Comparison of mean postoperative pain intensity (CI, 95%) between men and women on the verbal numerical rating scale.

No differences between men and women were found in the mean postoperative pain intensity measured using the vNRS (p > 0.05) (see Fig. 3).

Men had a higher mean body surface, 1.92 (SD = 0.20) than women, 1.69 (SD = 0.16), (p < 0.001). Men required an average of 7.247 mg of morphine hydrochloride for each unit of body surface area and women required 6.254 mg for the same unit of body surface area. There were no significant differences in analgesic requirements in milligrams of morphine hydrochloride, between men and women, to keep their pain level below 4 on the vNRS (p = 0.241).

## Discussion

Thoracic surgery requiring sternotomy can be very painful, with movement, coughing, and respiratory physiotherapy exacerbating the pain^18,19^. In this study, patients experienced a decrease in the mean intensity of pain over the course of successive shifts in the first 48 h of the postoperative period, maintaining values within the range of moderate to mild pain (mean level of pain below 4 on the vNRS). Only at day one the afternoon mean pain score was > 4, coinciding with the first hours after surgery.

In our study population, higher levels of preoperative SA proved to have a negative impact on the immediate postoperative period, increasing the analgesic requirements needed to keep patients’ pain below 4 on the vNRS. This finding offers an opportunity for intervention, as SA is a variable that can be modified via effective intervention to reduce preoperative anxiety and influence postoperative pain. Although several techniques have been identified for reducing anxiety, it is unclear which is the most effective. Interventions that have proved effective in reducing preoperative anxiety include ‘orientation tours’, in which patients visit the operating theatres and several inpatient units, where healthcare professionals explain their functioning and patients are able to share experiences with hospitalised patients before undergoing surgery^20^.

A number of studies have explored the role of psychological interventions in reducing pain intensity in patients undergoing cardiac surgery, obtaining no conclusive findings as to their effectiveness in reducing postoperative pain, although most of these studies did not consider postsurgical analgesic requirements^21^. One of the strengths of our study is the fact that it does not compare preoperative anxiety with postoperative pain itself but with the analgesic requirements needed to keep postoperative pain in the mild range, below 4 on the vNRS. To assess the analgesic needs of patients in order to keep pain intensity for mild to moderate pain (vNRS ≤ 4) in such painful surgery, could be considered as an indicator of adequate pain management in these patients. On the one hand analgesics need to be administrated regularly for moderate–severe pain and, on the other, patients need to be encouraged to communicate their pain with nurses.

Given the multifactorial nature of pain, extracorporeal circulation time and the type of surgery were taken into account as influential factors. The literature reports that patients undergoing cardiac surgery with the use of extracorporeal circulation report a slightly greater intensity of pain than those in whom extracorporeal circulation is not used, as it is associated with the induction of the systemic inflammatory response syndrome^6^. Besides, pain scores have been related to the type of surgery, some previous studies identified that patients having coronary artery bypass grafting **(**CABG) with Left Internal Mammary Artery (LIMA) and valvular surgery were more likely to have higher levels of pain than those having CABG without LIMA^22^.

In our study population, no differences in preoperative anxiety were found between men and women, despite some studies observing higher levels of anxiety among women^23^. There was also a decrease in analgesic requirements for every extra year of age, other studies have confirmed that the pain threshold rises with age^24^. Since our findings on sex refer specifically to postsurgical pain measured via analgesic requirements, they may differ from studies concluding that women are more sensitive to chronic pain^13^. However, our results corroborate recent studies finding increasing evidence that sex specific differences in pain are not clear. Even more controversial is the effect of sex on opioid consumption after surgery. Although morphine seems to be more effective in women compared to men, clinical trials investigating sex differences of perioperative morphine consumption showed conflicting results, in this way, female sex is not considered a risk factor for pain after surgery^25^.

Exploring associations between preoperative anxiety, perceptions of postoperative pain, and analgesic requirements may help us to plan pain management for patients undergoing cardiac surgery and deliver person-centred care. Nursing interventions could be designed to reduce postoperative pain by including an evaluation of state anxiety in the preoperative protocol for cardiac surgery. These results should be validated in other cohorts and hospital settings.

### Limitations

Limitation is linked to the use of a convenience sample of patients undergoing cardiac surgery in a specific hospital, which helped with meeting the study objectives but makes it difficult to generalise the results. Besides, preoperative pain has not been measured, which could be a risk of bias due to a possible association with postoperative pain.

## Conclusions

Postoperative analgesia requirements increase with higher levels of preoperative anxiety and decrease with each additional year of age, regardless of sex. Therefore, higher levels of preoperative state anxiety and lower age appear to act as predictors of greater analgesia requirements in cardiac surgery patients. Nursing interventions could be designed to reduce postoperative pain by including an evaluation of state anxiety in the preoperative protocol for cardiac surgery. These results should be validated in other cohorts and hospital settings.

## Data Availability

The datasets used and/or analysed during the current study are available from the corresponding author on reasonable request.

## Abbreviations

STAI: The State-Trait Anxiety Inventory
SA: State anxiety
TA: Trait anxiety
vNRS: Verbal numerical rating scale
